# Supplementary material for: Dynamical Screening of Local Spin Moments at Metal–Molecule Interfaces
Source: ACS Nano. 2023 Mar 7;17(6):5974–83. doi: 10.1021/acsnano.3c00247 (PMC10062023; doi:10.1021/acsnano.3c00247)
Supplement: Supplementary file 1 — nn3c00247_si_001.pdf [file nn3c00247_si_001.pdf]

Supporting information: Dynamical screening of local spin moments at metal-molecule interfaces

Sumanta Bhandary,*,† Emiliano Poli,‡ Gilberto Teobaldi,‡,¶ and David D. O'Regan†

†*School of Physics and CRANN Institute, Trinity College Dublin, The University of Dublin, Dublin 2, Ireland*

‡*Scientific Computing Department, STFC UKRI, Rutherford Appleton Laboratory, Didcot OX11 0QX, United Kingdom*

¶*School of Chemistry, University of Southampton, Highfield, Southampton SO17 1BJ , United Kingdom*

E-mail: sumanta.bhandary@tcd.ie

1 Electronic structure simulations

We performed our first-principles calculations using density functional theory (DFT) as implemented within the Vienna ab-initio Simulation Package (VASP).[1] To obtain parameters for the many-body calculations, that take into account dynamical correlations effects, specifically, we performed non-spin polarised DFT calculations using the DFT-relaxed structures. In the following, we discuss how the ab-initio parameters are extracted to describe a correlated sub-space within the Anderson Impurity Model as well as the details of many-body simulations.

1.1 Many-body effects: DFT++

To obtain a realistic description of screening of the local magnetic moments of the molecules at metal contact, we employed a combined approach: density functional theory (DFT) plus many-body technique within multi-orbital Anderson impurity model (AIM). The method is often referred to as the DFT++ method.[2]

The first realistic description of the molecule-surface hybrids is obtained within DFT. This allows us to extract *ab-initio* parameters to describe a correlated sub-space, i.e., the impurity, corresponding to the TM-3d multiplet, which is coupled to an electron bath, corresponding to the rest of the system: phthalocyanine ring plus the Cu(111) surface, through a retarded hybridization function. In the AIM, the impurity Hamiltonian is supplemented with a Coulomb interaction, and the concomitant many-body problem is solved numerically. The complete Hamiltonian of the AIM can be written as

$$\begin{aligned} H = \sum_{ij} \epsilon^d_{ij} d^\dagger_{i\sigma} d_{j\sigma} + \frac{1}{2} \sum_{ijkl} \sum_{\sigma\sigma'} U_{ijkl} d^\dagger_{i\sigma} d^\dagger_{j\sigma'} d_{l\sigma'} d_{k\sigma} \\ + \sum_{im} \sum_{\sigma} (V_{im} c^\dagger_{m\sigma} d_{i\sigma} + h.c.) + \sum_{m} \sum_{\sigma} \epsilon^b_m c^\dagger_{m\sigma} c_{m\sigma}, \end{aligned} \tag{S1}$$

where $d_{i\sigma}$ ($d^\dagger_{i\sigma}$) are the annihilation (creation) operators of an electron in impurity (TM-

$3d$) orbital i with spin σ, and $c_{m\sigma}(c^{\dagger}_{m\sigma})$ denote the annihilation (creation) operators of an electron in bath orbital m with spin σ and energy ϵ^{b}_{m}. V_{im} denotes the coupling between the TM-3d and the bath orbitals, ϵ^{d}_{ij} describes the crystal field matrix and U_{ijkl} represents the full Coulomb tensor within the TM-3d multiplet. The rotationally-invariant Coulomb interaction is parametrized via the Slater radial integrals[3,4] F^0, F^2, and F^4, such that $U = F^0$ and $J = \frac{1}{14}(F^2 + F^4)$, with the ratio $F^4/F^2 = 0.625$, yielding a spherically symmetric tensor.[5,6] In all of our calculations, we have used U= 4.0 eV and J=1.0 eV. We note that when the spherical symmetry is lifted in presence of a crystal field, the effect can be taken into account, e.g., within the constrained random phase approximation.[7] In n our analysis, we do not expect this neglected effect to change our main conclusions.

For the numerical solution of the many-body problem, we have used a continuous-time hybridization-expansion quantum Monte Carlo (QMC) solver as implemented in w2dynamics program package.[8] The dynamical nature of the hybridization function is fully taken into consideration within the QMC algorithm. We emphasize that discretised Hamiltonian approaches, such as exact diagonalisation, are inadequate in the case of molecules adsorbed on metallic surfaces due to a broad nature of the hybridization function. The double counting correction term is implemented by fixing the chemical potential corresponding to the occupation of the impurity orbitals, $n = \sum_{i\sigma}\langle n_{i\sigma}\rangle$. In this work, the total impurity occupations considered as, n, are 2.57e (TiPc), 3.47e (VPc), 4.67e (CrPc), 5.81e (MnPc), 6.62e (FePc), 7.66e (CoPc), 8.13e (NiPc), and 9.32e (CuPc). We note that the off-diagonal elements of the Hamiltonian and the hybridization functions are significantly small and have been neglected to avoid technical complications, and this approximation provides an orbital-diagonal QMC self-energy.

1.2 Embedding the transition metal(TM)-atom: Hybridization function

In this section, we explain how the TM atoms embed in the phthalocyanine plus Cu-surface environment, we calculate the *ab-initio* hybridization function from the Kohn-Sham Green's function, G_{KS}, in the Lehmann representation:

$$G_{KS}(\omega) = \sum_{nk} \frac{|\psi_{nk}\rangle \langle\psi_{nk}|}{\omega + i\delta - \epsilon_{nk}}, \tag{S2}$$

where ψ_{nk} and ϵ_{nk} are the Kohn-Sham eigenstates and eigenvalues for band n and reciprocal-space point k, while δ is an infinitesimal broadening, meaning that G_{KS} is the retarded propagator. The G_{KS} is then projected down to form the local (or the impurity) propagator G_0, This projection is defined using atom-centered, localized orbitals χ_i. In this basis, the local Green's function is written as

$$G_0^{ij}(\omega) = \sum_{nk} \frac{\tilde{P}^i_{nk}(\tilde{P}^j_{nk})^*}{\omega + i\delta - \epsilon_{nk}}, \tag{S3}$$

where $P^i_{nk} = \langle\chi_i|\psi_{nk}\rangle$ are projection matrices, which are ortho-normalized according to

$$\tilde{P}^i_{nk} = \sum_j [O(k)]^{-1/2} P^j_{nk}, \tag{S4}$$

using the effective overlap operator

$$O_{ij}(k) = \sum_n P^i_{nk}(P^j_{nk})^*. \tag{S5}$$

Finally, the hybridization function is calculated from the local impurity Green's function from the expression

$$\Delta_{ij}(\omega) = \left[\omega + i\delta\right]\delta_{ij} - \epsilon^d_{ij} - \left[G_0^{-1}\right]_{ij}(\omega). \tag{S6}$$

At this point, one can obtain the impurity-bath coupling matrix elements by fitting the

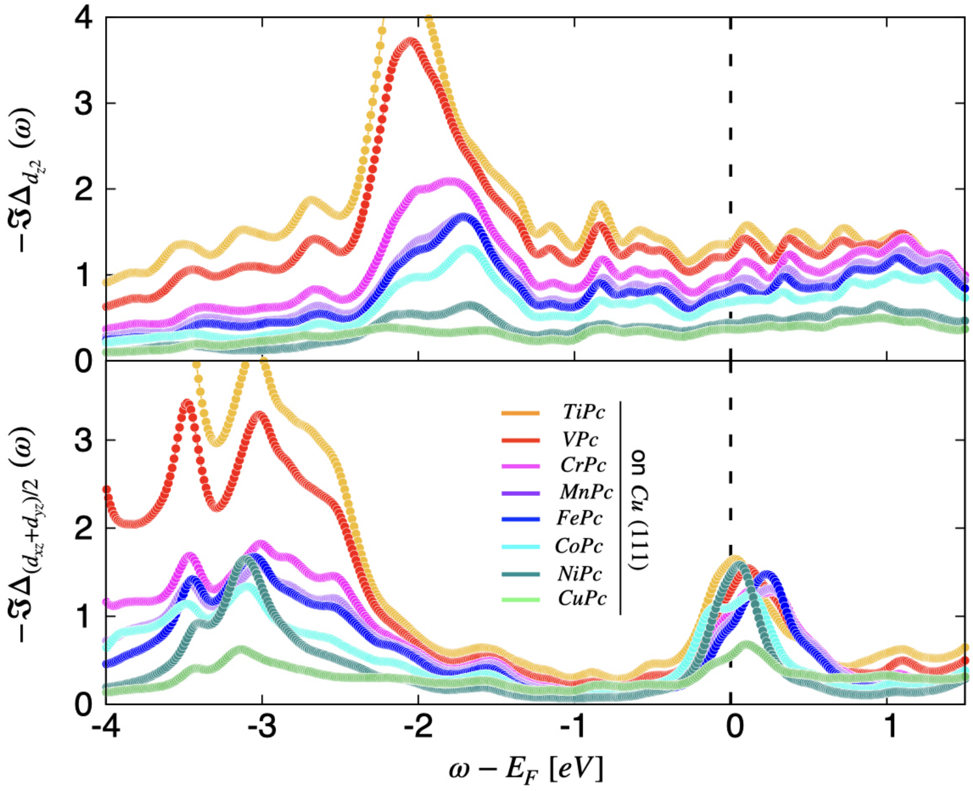

Figure S1: Hybridization function (Δ) for the out-of-plane orbitals for different TMPcs on Cu(111). (a) Imaginary part of $\Delta_{d_{z^2}}$ for TMPc molecules reflecting the metallic hybridization. (b) The same but for the average of the d_{xz} and d_{yz} orbitals. A broad Δ around the Fermi energy signifies the coupling of the TM atoms with the Cu surface states.

hybridization function with the following:

$$\Delta_{ij}(\omega) = \sum_m \frac{V_{im}V_{mj}}{\omega + i\delta - \epsilon_m^b}, \tag{S7}$$

where $i, j = \{d_{xy}, d_{yz}, d_{z^2}, d_{xz}, d_{x^2-y^2}\}$ are the TM-3d orbitals, ϵ_m^b the energies of the molecular orbitals of the ligands or the so-called bath orbitals, which include states from both the phthalocyanine ring and the Cu(111) surface underneath, and V_{im} their coupling. The crystal field energies, ϵ_{ij}^d, are obtained from Δ_{ij} for $\omega \to \infty$. Both of these parameter sets can be used in discrete Hamiltonian solvers for Anderson Impurity Model (AIM), such as Exact Diagonalization. However, in the systems under present study, where that the

hybridization is energetically broad, discrete Hamiltonian methods are inadequate.

We note that, if the projected TM-3d orbitals are not effectively orthonormal (i.e., if $\epsilon^d_{i\neq j} \neq 0$), then the hybridization function includes also off-diagonal terms. In the present scenario, $\Delta_{i\neq j} \ll \Delta_{ii}$ for any pair (i, j). We show the diagonal elements of the hybridization function $\Delta_{ii}(\omega)$ in Fig. 2 in the main text, as well as in Fig. S1. In Fig. S1, we depict the hybridization function of the out-of-plane orbitals i.e., the d_{z^2} , d_{xz} and d_{yz} orbitals for all of the TMPc molecules on the Cu surface. We note that the hybridization of the d_{xz} and d_{yz} orbitals are quasi-degenerate, hence we plot the average. The $\Im\Delta_{\mathrm{d}_{z^2}}$ is broad around the Fermi level in comparison with the $\Im\Delta$ of the d_{xz} and d_{yz} orbitals, signifying direct hybridization with the broad metallic states of the Cu surface. The strength of the hybridization, particularly of the d_{z^2} orbital, is stronger for early TM ions and gradually weakens for the later TM ions in the series. This reflects the the gradual increase of covalent radius as the atomic number decreases.

To show how the hybridization changes in TMPc molecules, we obtained the coupling strengths V_{im} (or simply V_i) corresponding to the axial bonds between the $\mathrm{d}_{x^2-y^2}$ orbital and predominantly the N-2p orbitals of the phthalocyanine ring. We also obtained the bath energy, ϵ^b_m, corresponding to the strongest peak in the $\Im\Delta_{\mathrm{d}_{x^2-y^2}}$ (as seen in Fig. 2 in the main text). The values are presented in Table 1.

Table 1: The coupling strengths (V_i) and the bath energies (ϵ^b) corresponding to the strongest peak in the hybridization function for the $\mathrm{d}_{x^2-y^2}$ orbital of all TMPc molecules.

Parameters [eV]	TMPc molecules on Cu(111)							
	TiPc	VPc	CrPc	MnPc	FePc	CoPc	NiPc	CuPc
$V_{\mathrm{d}_{x^2-y^2}}$	3.62	3.58	3.61	3.45	3.44	3.21	3.02	2.54
ϵ^b	-2.49	-2.53	-2.53	-2.66	-2.66	-3.02	-3.26	-2.91

2 Molecular adsorption

Table 2: Adsorption energies of the TMPc molecules analysed on Cu(111) surfaces without considering the vdW correction.

TMPc	Adsorption energies [eV]							
	TiPc	VPc	CrPc	MnPc	FePc	CoPc	NiPc	CuPc
Energy	-0.62	-0.43	-0.22	-0.26	-0.12	-0.37	-0.08	-0.14

The absorption energies reported in Table 2 without the D2 vdW energy corrections present a different picture from the one reported in Table 1 in the main text. The adsorption values are equal to or less than 0.4 eV (except for the Ti case). Such an energy range is typical of the physisorption regime. It has to be noted, however, that the PBE functional tends to underestimate the absorption energy at metallic surfaces[9–11] while the PBE-D2 approach tends to overestimate them.[12] We thus expect the TMPc to be in a hybrid absorption regime between chemisorption and physisorption. The structural and atomic charge analysis in the main text and in the paragraph below support such hypothesis. In fact, while the TMPc molecules are distorted when put in contact with the Cu(111) surface, and a significant hybridization of the out-of-plane 3d orbitals with the metallic states of Cu(111) surface is observed (Figure 2c, main text), no visible bond breaking or rearrangement is found in our analysis. In addition, while we observed a noticeable charge transfer between the Cu(111) surface and the TMPc (Figure S2), the electronic charge redistributed is always less than 1 e^- in total. These charge transfers are redistributed thanks to delocalization throughout the whole organic framework of the TMPc, causing distortion but not bond breaking.

In the following we discuss the changes in the TMPc molecules due to adsorption in comparison with the free TMPc molecules.

2.1 Structural changes

Below, we compare the change in the TM-N bond lengths of free and adsorbed molecules, as those are crucial for the ligand field and hence the spin-state. The structural distortions in connection with the non-planarity are discussed in the main text (Fig. 1).

Table 3: The TM-N bond lengths in free and adsorbed molecules

TMPc	TM-N bond lengths [Å]							
	TiPc	VPc	CrPc	MnPc	FePc	CoPc	NiPc	CuPc
Free	2.01	1.98	1.98	1.94/1.95	1.93	1.92	1.91	1.96
Adsorbed	2.04/2.05	2.01/2.02	1.98/1.99	1.95/1.96	1.93	1.91/1.92	1.91/1.92	1.98

In general, adsorption breaks the D_{4h} symmetry of the isolated molecules due to local structural mismatch. Crucial to the ligand field, the TM-N bond lengths also change due to the adsorption. Molecules embedding early TM ions show a larger expansion of the TM-N core, while as the surface molecule distance is increased in the later part of the TM series, the TM-N bond distances appear similar to the free molecule counterparts.

2.2 Bader charge analysis

Bader charge analysis was carried out on the basis of the total charge density, accounting for both the electronic and ionic core charges. We performed the charge analysis on both the full systems (TMPc/Cu(111)) and the TMPc molecules in the gas phase in order to understand the charge transfer behaviour at the interface. Upon adsorption on the Cu-substrate, all the TMPc molecules acquire electronic charge from the surface as shown in Figure S2 (upper panel). In particular, the central transition metal ions are less positively charged in all the structures with respect to their gas phase counterparts, with the exception of VPc. In relation to the molecule/surface distortion analysis it can be noticed that the maximum amount of charge is transferred to the TMPc that are neither strongly or weakly adsorbed, as in the Cr, Mn and Fe cases where the distance between the metal ions and the Cu surface is of $\sim$ 2.6Å. Considering the different atom types comprising the organic framework of the

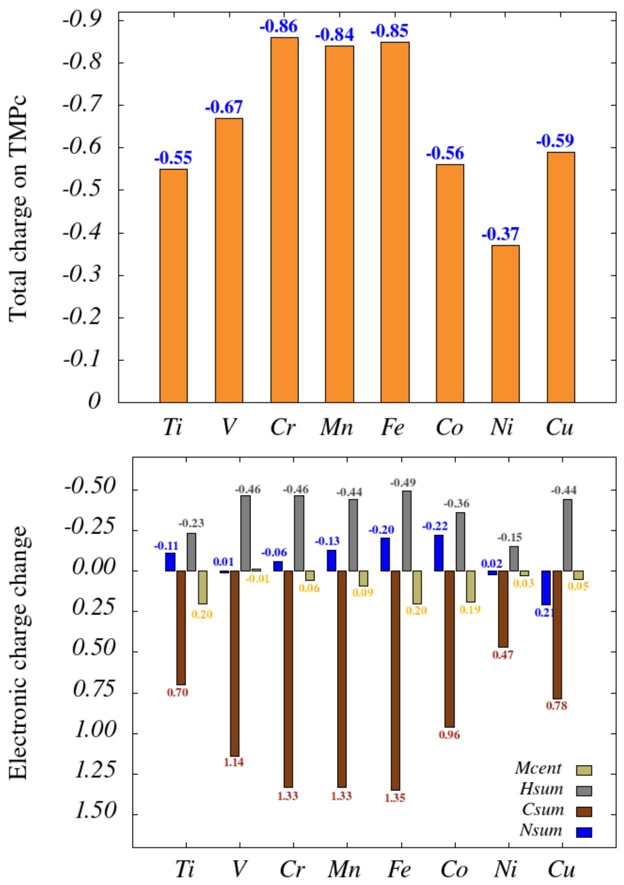

Figure S2: Net charge transferred from the Cu(111) surface to the TMPc molecule (upper panel) and total electronic charge changes per atom type upon TMPc absorption (lower panel). A positive value in the lower panel means an increment in the electronic charge (i.e., less positive atom). Vice versa a negative value indicates a depletion of electronic charge (i.e., more positive atom).

phtalocyanines (see Figure S2 bottom panel) no straightforward trend can be recognized. In general the carbon atoms tend to become less positive (i.e., acquire electronic charge) while hydrogen and nitrogen atoms decrease their electronic charge. However, there are exceptions (i.e., VPc, CoPc, and NiPc) where the nitrogen atoms become more negatively charged. For each TMPc, a different charge accumulation/depletion pattern with respect to the gas phase case can be observed for the organic framework of the molecules.

3 Magnetism and screening in adsorbed TMPc molecules

3.1 Double occupation, charge and spin fluctuations

In Fig. S3, we present the double occupation for like and opposite spins (first and second row, respectively), charge and spin fluctuations (third and the fourth row) for the TMPc molecules on Cu(111) that are not presented in the main paper, i.e., TiPc, MnPc, CoPc, and CuPc.

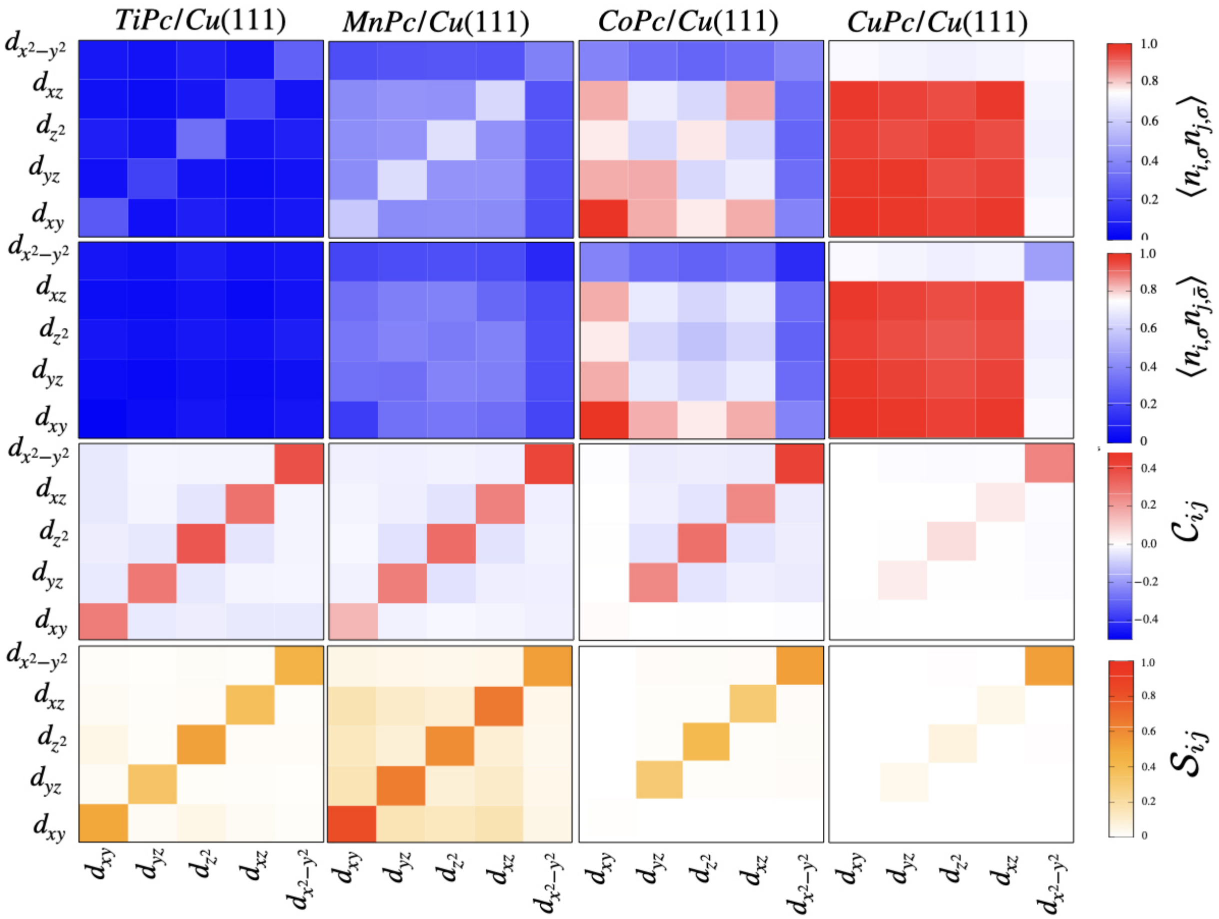

Figure S3: Heat-map representations of the double occupation $\langle n_{i,\sigma} n_{j,\sigma} \rangle$ for like spins (1st row), double occupation $\langle n_{i,\sigma} n_{j,\bar{\sigma}} \rangle$ for opposite spins (2nd row), charge- (3rd row) and spin-fluctuations (4th row) in TMPc molecules (TM= Ti, Mn, Co, Cu) on Cu(111).

We see significantly enhanced charge fluctuations and weakened spin fluctuations in the d_R subspace in TiPc, MnPc, and CoPc. As we discussed in the main paper, both of these

effects significantly screen the local spins in the same subspace, which is responsible for the local magnetic moment in these molecules. We, therefore, see a large deviation in the long-time moments from the corresponding instantaneous moments, as presented in Fig. 3 in the main paper. The scenario is different in CuPc. There the d_R subspace is completely filled, yielding non-existent charge and spin fluctuations (i. e. $\mathcal{C}_{ij}$ and $\mathcal{S}_{ij} \approx 0$). In Fig. S3, we observe a relatively strong ferromagnetic spin fluctuation in the $d_{x^2-y^2}$ orbital, which is the origin of the local moment in CuPc.

In Fig. S4, we show the orbital-diagonal terms of spin susceptibility, $\chi_{ii}(\tau)$, of all the TMPc molecules, studied in this paper. As we discussed in the main paper, the gaps between

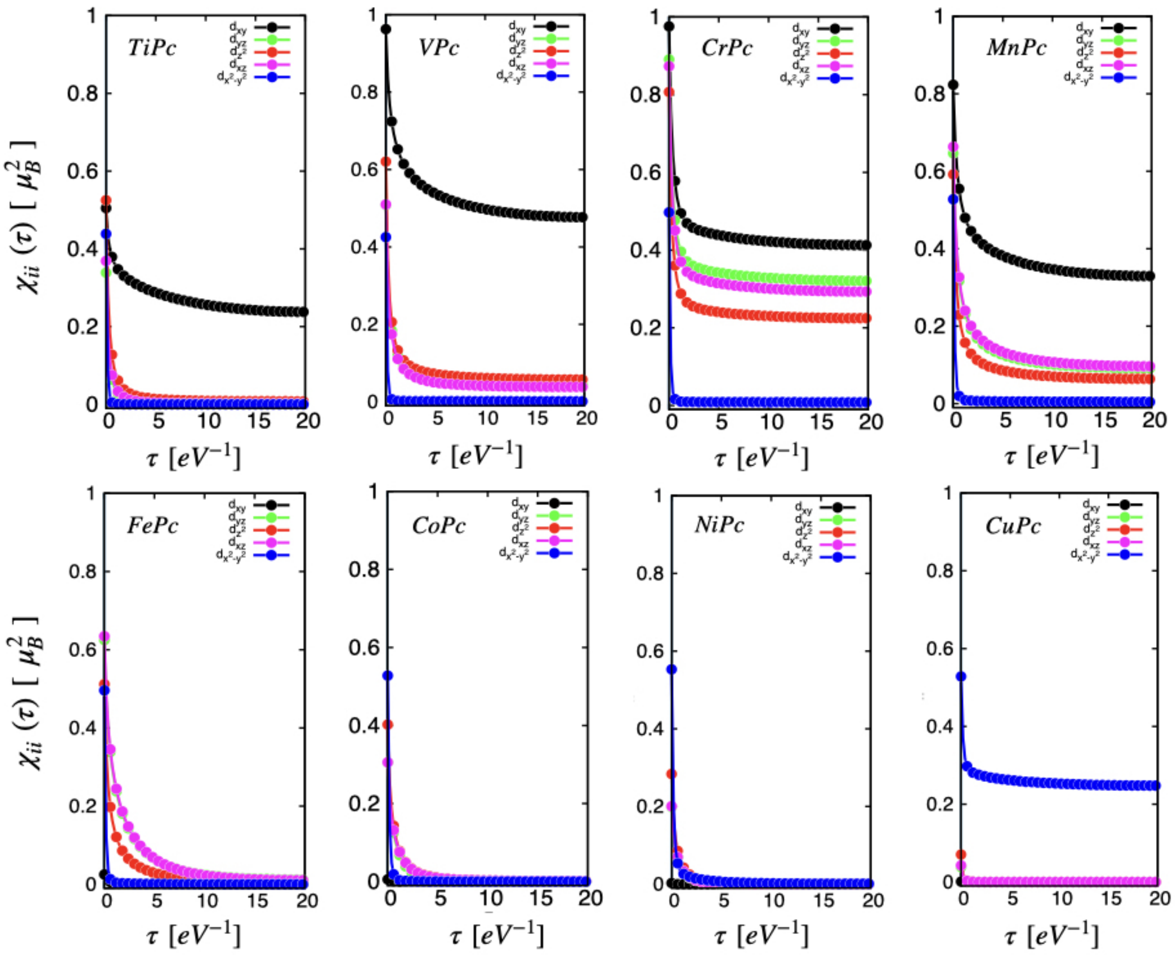

Figure S4: Orbital resolved spin susceptibility (χ_{ii}) in imaginary time τ for all TM ions in TMPc/Cu(111) systems.

the $\chi_{ii}(\tau = 0)$ and $\chi_{ii}(\tau = \beta/2)$ mark the screening of the spins in those orbitals. In Fig. S4, we show the different degree of such screening in different $3d$ orbitals within the same TMPc molecule, as well as the variation in different TMPc molecules adsorbed on Cu(111) surface. However, we note that in CuPc the scenario is different from the rest of the molecules. Unlike in other molecules, there both $\chi(0)$ and $\chi(\beta/2)$ are comprised solely of the $d_{x^2-y^2}$ orbital contribution, signifying that the instantaneous and the screened local moments have a $d_{x^2-y^2}$ origin.

3.2 Temperature dependence of dynamical screening

We discuss the temperature dependence of the dynamical processes that leads to screening of the local moments in TMPc molecules. We considered FePc/Cu(111) for this specific study. In Fig. S5(a), we plot the instantaneous (S_{inst}) and screened (S_{scr}) spin moments of the FePc molecule (adsorbed on Cu(111)) at temperatures ranging from 30K to 290K. We note that here we performed the AIM calculations in a reduced orbital space comprising of the d_{xz}, d_{yz}, and d_{z^2} orbitals of the Fe atom. Such an approximation is justified because in FePc, the d_{xy} orbital is completely filled and, hence, does not contribute to the local moment. As discussed in the main paper, the spin on the $d_{x^2-y^2}$ orbital is screened completely; therefore, as far as the screened moment is concerned, $d_{x^2-y^2}$ does not have any contribution. Nonetheless, the instantaneous moment has a significant contribution from the $d_{x^2-y^2}$ orbital. To elucidate this contrast, in Fig. S5(a) we compare the S_{inst} and S_{scr} as calculated from the full 5 $3d$-orbital AIM calculations, against those from the reduced-space 3 $3d$-orbital AIM calculations, all at 290K. One can see that the S_{inst} from a 5 orbital calculation differs from the same for 3 orbital calculation, while the screened moments do not substantially differ.

From these results, we also see that the S_{inst} remain constant in the temperature range, while the screened moments show a clear temperature dependence. We note that even at 290K, the long-time moment is strongly suppressed due to the dynamical screening, as also discussed in the main paper. Upon lowering the temperature, the S_{scr} (almost) linearly

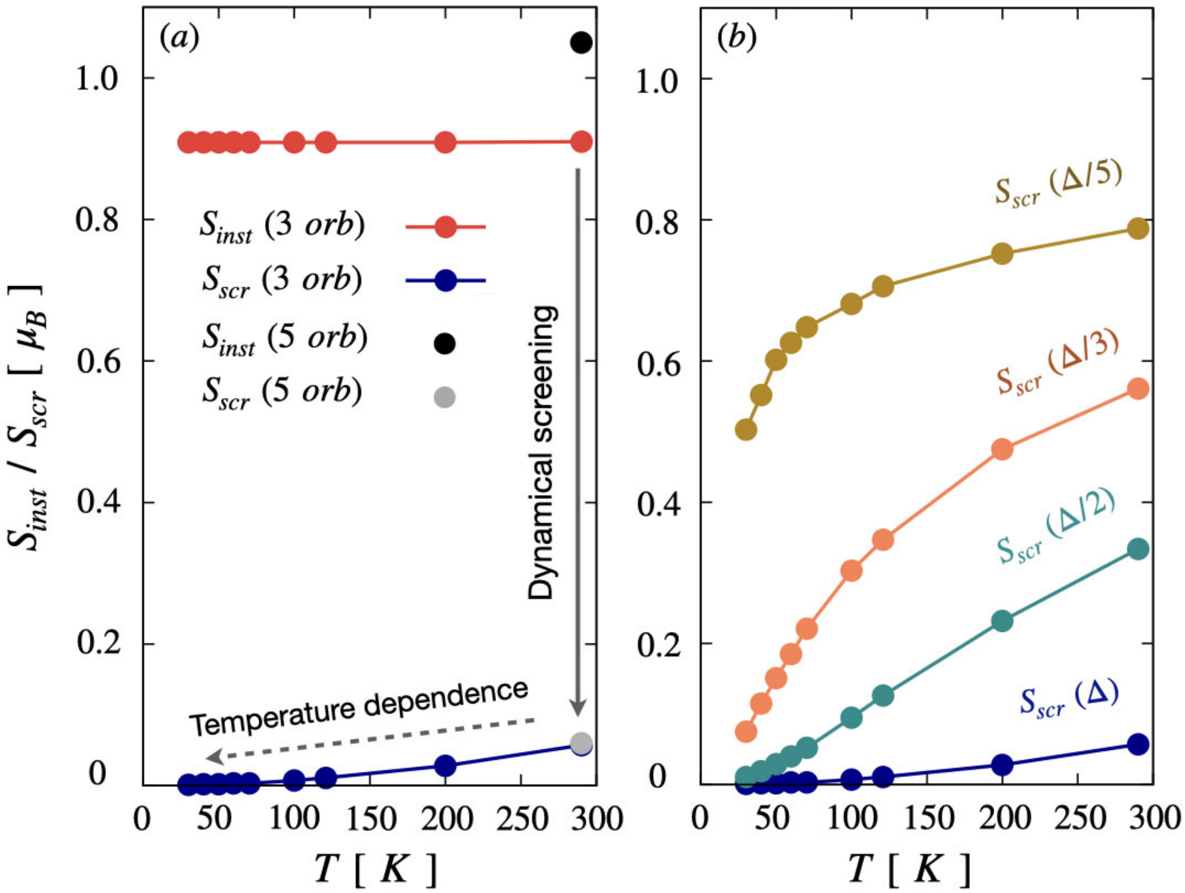

Figure S5: (a) Temperature dependence of the local spin moment in FePc/Cu(111), obtained within 3-orbital AIM. (b) Temperature-dependent behavior upon reduction of the hybridization strengths.

reaches approximately 0 μ_B. This is consistent with what is expected in a metallic system, namely that at a long enough time, a complete screening takes place at low-T due to strong fluctuations of the local moment induced by the high electron mobility.

We finally elucidate the role of hybridization, hence the electron mobility, in the temperature-dependent screening, in Fig. S5(b). Here, we plot the temperature dependence of the S_{scr} upon an artificial lowering of the hybridization, Δ. At $\Delta/2$, the behaviour is linear-like although screening is significantly less, particularly at the higher temperature regime. If further decreased, at $\Delta/3$, the linear behavior is lifted and local spin is not quenched at the lowest temperature we can reach (30K), while at $\Delta/5$, the local moment is only partially

screened, retaining large S_{scr} even at that low temperature.

4 Magnetism and screening in free TMPc molecules

4.1 Effective local spins

We discuss the magnetic behavior of the free TMPc molecules in the absence of the surface underneath. We note that the structures of the free molecules are relaxed following the criteria mentioned in the main text (sec. Methods). The results provide 1) a direct comparison with the existing experimental results, and 2) insight into the role of hybridization in the dynamical screening process, in particular via the out-of-plane orbitals.

In Fig. S6, similarly as performed for the adsorbed molecules in the main text, we plot the total spins of the free TMPc molecules obtained with DFT+AIM and DFT+U calculations. The purple and the cyan bars depict the instantaneous (S_{inst}) and screened (S_{scr}) effective spin moments, respectively, while the green bars represent spin moments obtained in DFT+U.

One can immediately notice that, in general, the S_{inst} are atomic-like, mostly driven by inter-orbital Hund's coupling, and have similar values as the adsorbed TMPc molecules on Cu. It is noteworthy that, as discussed earlier, surface adsorption causes a structural change, and hence a change in the ligand field in the molecule, while simultaneously allowing an effective charge transfer. The effect of surface hybridization in the instantaneous local moments is minimal. However, in comparison with the adsorbed molecules, the long-time spin moments are significantly less screened. We obtained, $S_{scr} \approx 0.5\mu_{\mathrm{B}}$(TiPc), $1.3\mu_{\mathrm{B}}$(VPc), $1.6\mu_{\mathrm{B}}$(CrPc), $1.4\mu_{\mathrm{B}}$(MnPc), $1.0\mu_{\mathrm{B}}$(FePc), $0.5\mu_{\mathrm{B}}$(CoPc), $0.0\mu_{\mathrm{B}}$(NiPc), $0.2\mu_{\mathrm{B}}$(CuPc). Our obtained values are in good agreement with the existing experimental observations, which find: $1.3\mu_{\mathrm{B}}$(CrPc),[13] $1.5\mu_{\mathrm{B}}$(MnPc),[14–18] $1.0\mu_{\mathrm{B}}$(FePc),[19–23] $0.5\mu_{\mathrm{B}}$(CoPc),[24,25] $0.0\mu_{\mathrm{B}}$(NiPc), $0.5\mu_{\mathrm{B}}$(CuPc).[17,18,26] In these calculations, the double counting correction term is implemented by fixing the chemical potential corresponding to the occupation of the impurity

orbitals, $n = \sum_{i\sigma} \langle n_{i\sigma} \rangle$. The total impurity occupations considered as, n, are 2.38e (TiPc), 3.51e (VPc), 4.61e (CrPc), 5.73e (MnPc), 6.52e (FePc), 7.57e (CoPc), 8.50e (NiPc), and 9.26e (CuPc).

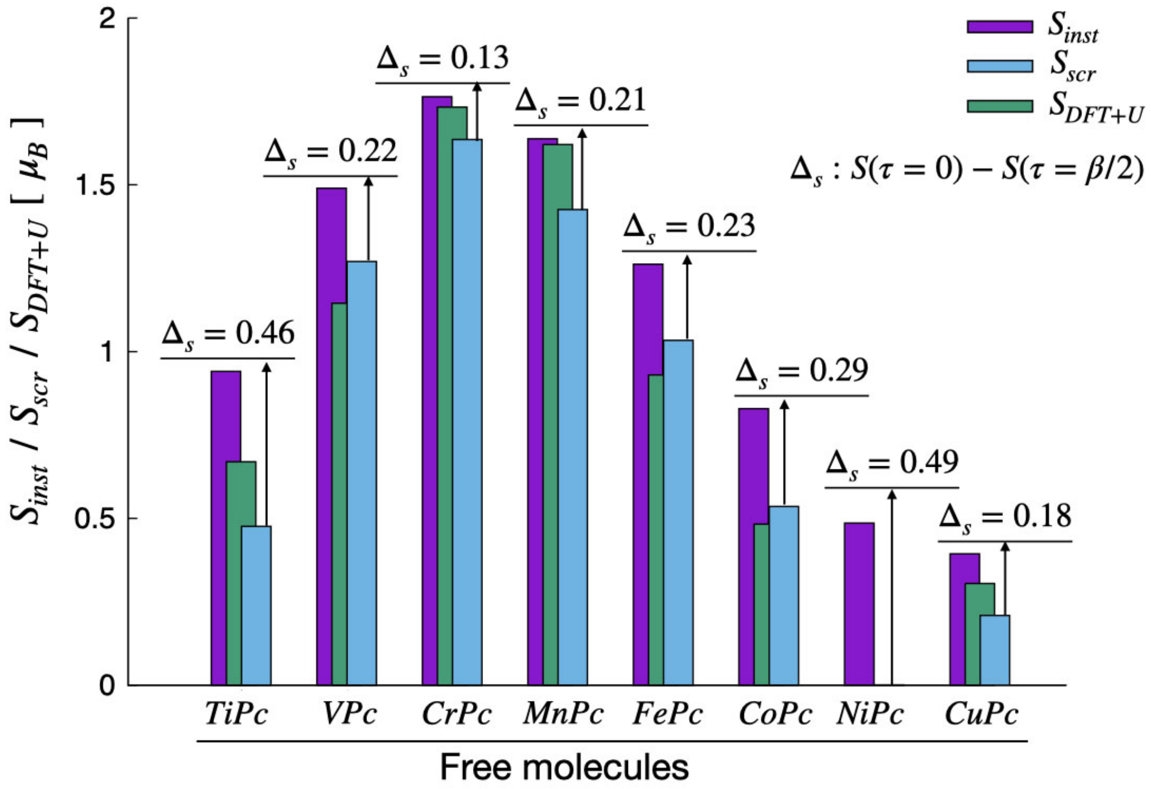

Figure S6: Dynamical screening of the effective moments in isolated TMPc molecules. Unscreened (purple), screened (cyan), and DFT+U effective spin moments (green) for TMPc molecules. Δ_s is the difference between the instantaneous and screened spin moments.

It is to be noted that, unlike in the case of adsorbed molecules, the DFT+U moments are very similar to the screened moments obtained in AIM. In absence of the surface induced valence fluctuation, the charge fluctuation is rather small. As discussed in the following subsection, one sees a flattening of $\chi(\tau)$ which reflects poor screening, and which is a signature of correlated insulators. In such a scenario, treating of static exchange and correlation effects at the level of DFT+U works reasonably well, albeit without providing insights on transient behaviour.

4.2 Spin susceptibility

In Fig. S7, we present the orbital resolved local spin-susceptibility, $\chi_{ii}(\tau)$, for the free TMPc molecules. Distinguishable from the adsorbed molecules (particularly the out-of-plane orbitals), one observes a flattening in $\chi_{ii}(\tau)$, which stems from poor screening in absence of the hybridization with the surface. In CrPc, all d_R orbitals contribute (almost) equally to

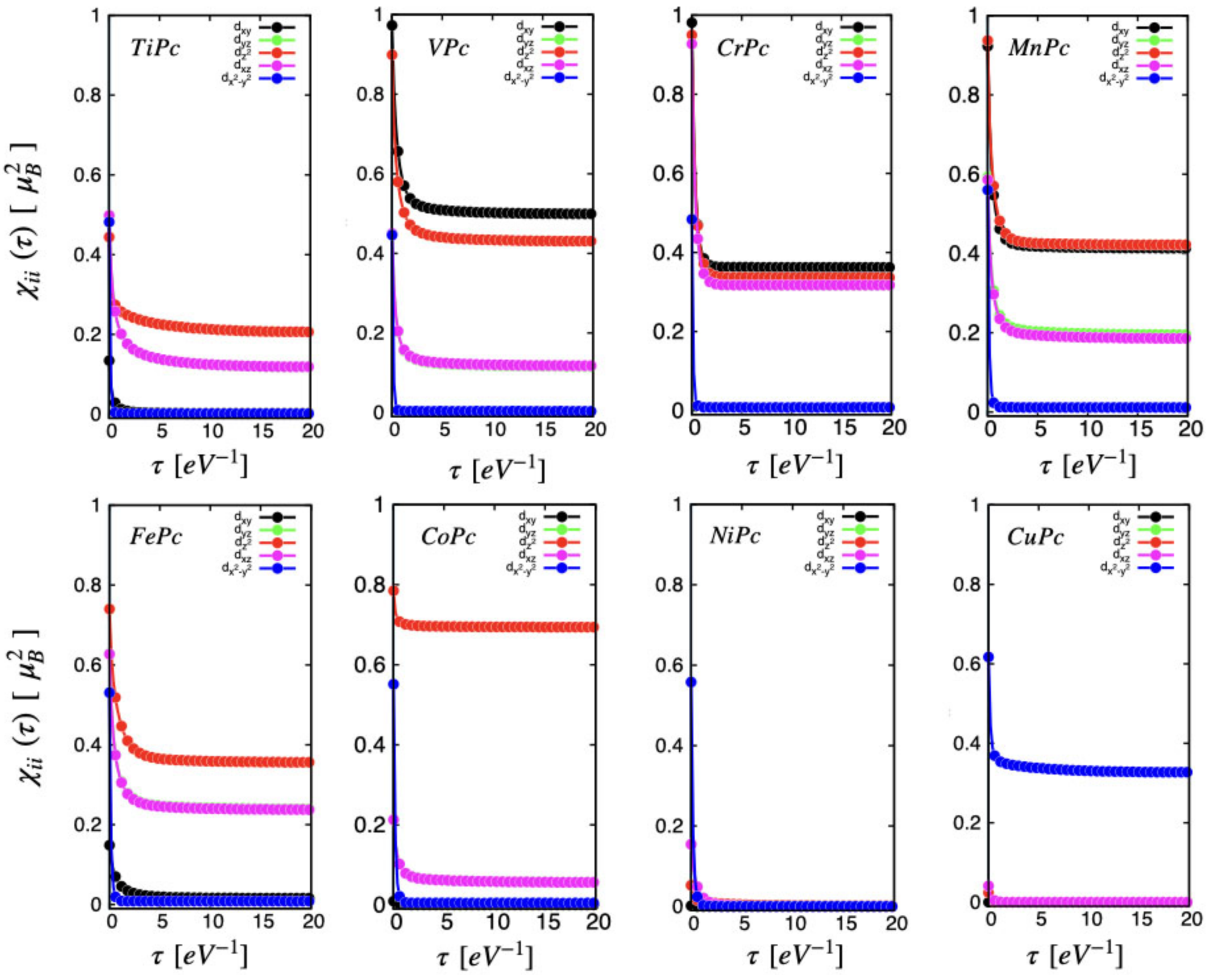

Figure S7: Orbital resolved spin susceptibility (χ_{ii}) in imaginary time τ for all TM ions in isolated TMPc molecules.

the long-time moment, signifying strong spin fluctuations dictated by Hund's physics. As expected, the Δ_s is the lowest among all TMPc molecules. Generally, in the absence of hybridization through the out-of-plane orbitals, the lack of screening results in significant

$\chi_{ii}(\beta/2)$ values, hence large persisting moments. Interestingly, in free CuPc, both S_{inst} and S_{scr} are comparable with the same in adsorbed CuPc. The magnetic moment in CuPc is carried out by the in-plane $d_{x^2-y^2}$ orbital, which is barely impacted by the surface induced screening, as also observed in experiments.[26,27]

References

(1) Kresse, G.; Furthmüller, J. Efficient iterative schemes for ab initio total-energy calculations using a plane-wave basis set. *Phys. Rev. B* **1996**, *54*, 11169–11186.

(2) Karolak, M.; Wehling, T. O.; Lechermann, F.; Lichtenstein, A. I. General DFT++ method implemented with projector augmented waves: electronic structure of SrVO3and the Mott transition in $Ca_{2-x}Sr_xRuO_4$. *Journal of Physics: Condensed Matter* **2011**, *23*, 085601.

(3) Slater, J. C. The theory of complex spectra. *Physical Review* **1929**, *34*, 1293.

(4) Slater, J. C. *Quantum Theory of Atomic Structure*; McGraw-Hill, New York, 1960.

(5) Karolak, M. *Ph.D. thesis, Universität Hamburg, (Hamburg)* **2013**,

(6) Valli, A.; Bahlke, M. P.; Kowalski, A.; Karolak, M.; Herrmann, C.; Sangiovanni, G. Kondo screening in Co adatoms with full Coulomb interaction. *Phys. Rev. Research* **2020**, *2*, 033432.

(7) Jacob, D. Towards a fullab initiotheory of strong electronic correlations in nanoscale devices. *Journal of Physics: Condensed Matter* **2015**, *27*, 245606.

(8) Wallerberger, M.; Hausoel, A.; Gunacker, P.; Kowalski, A.; Parragh, N.; Goth, F.; Held, K.; Sangiovanni, G. w2dynamics: Local one- and two-particle quantities from dynamical mean field theory. *Computer Physics Communications* **2019**, *235*, 388–399.

(9) Yuan, D.; Liao, H.; Hu, W. Assessment of van der Waals inclusive density functional theory methods for adsorption and selective dehydrogenation of formic acid on Pt(111) surface. *Phys. Chem. Chem. Phys.* **2019**, *21*, 21049–21056.

(10) Carrasco, J.; Liu, W.; Michaelides, A.; Tkatchenko, A. Insight into the description of van der Waals forces for benzene adsorption on transition metal (111) surfaces. *The Journal of Chemical Physics* **2014**, *140*, 084704.

(11) Tonigold, K.; Groß, A. Dispersive interactions in water bilayers at metallic surfaces: A comparison of the PBE and RPBE functional including semiempirical dispersion corrections. *Journal of Computational Chemistry* **2012**, *33*, 695–701.

(12) Al-Saidi, W. A.; Feng, H.; Fichthorn, K. A. Adsorption of Polyvinylpyrrolidone on Ag Surfaces: Insight into a Structure-Directing Agent. *Nano Letters* **2012**, *12*, 997–1001.

(13) Elvidge, J. A.; Lever, A. B. P. 245. Metal chelates. Part II. Phthalocyanine–chromium complexes and perpendicular conjugation. *Journal of the Chemical Society (Resumed)* **1961**, 1257–1265.

(14) Barraclough, C. G.; Martin, R. L.; Mitra, S.; Sherwood, R. C. Paramagnetic Anisotropy, Electronic Structure, and Ferromagnetism in Spin S=3/2 Manganese(II) Phthalocyanine. *The Journal of Chemical Physics* **1970**, *53*, 1638–1642.

(15) Mitra, S.; Gregson, A. K.; Hatfield, W. E.; Weller, R. R. Single-crystal magnetic study on ferromagnetic manganese(II) phthalocyaninate. *Inorganic Chemistry* **1983**, *22*, 1729–1732.

(16) Williamson, B. E.; VanCott, T. C.; Boyle, M. E.; Misener, G. C.; Stillman, M. J.; Schatz, P. N. Determination of the ground state of manganese phthalocyanine in an argon matrix using magnetic circular dichroism and absorption spectroscopy. *Journal of the American Chemical Society* **1992**, *114*, 2412–2419.

(17) Heutz, S.; Mitra, C.; Wu, W.; Fisher, A.; Kerridge, A.; Stoneham, M.; Harker, A. Gardener, J.; Tseng, H.-H.; Jones, T.; Renner, C.; Aeppli, G. Molecular Thin Films: A New Type of Magnetic Switch. *Advanced Materials* **2007**, *19*, 3618–3622.

(18) Kroll, T.; Kraus, R.; Schönfelder, R.; Aristov, V. Y.; Molodtsova, O. V.; Hoffmann, P.; Knupfer, M. Transition metal phthalocyanines: Insight into the electronic structure from soft x-ray spectroscopy. *The Journal of Chemical Physics* **2012**, *137*, 054306.

(19) Dale, B. W.; Williams, R. J. P.; Johnson, C. E.; Thorp, T. L. S=1 Spin State of Divalent Iron. I. Magnetic Properties of Phthalocyanine Iron (II). *The Journal of Chemical Physics* **1968**, *49*, 3441–3444.

(20) Barraclough, C. G.; Martin, R. L.; Mitra, S.; Sherwood, R. C. Paramagnetic Anisotropy, Low Temperature Magnetization, and Electronic Structure of Iron(II) Phthalocyanine. *The Journal of Chemical Physics* **1970**, *53*, 1643–1648.

(21) Coppens, P.; Li, L.; Zhu, N. J. Electronic ground state of iron(II) phthalocyanine as determined from accurate diffraction data. *Journal of the American Chemical Society* **1983**, *105*, 6173–6174.

(22) Evangelisti, M.; Bartolomé, J.; de Jongh, L. J.; Filoti, G. Magnetic properties of α-iron(II) phthalocyanine. *Physical Review B* **2002**, *66*, 144410.

(23) Miedema, P. S.; Stepanow, S.; Gambardella, P.; Groot, F. M. F. d. 2p x-ray absorption of iron-phthalocyanine. *Journal of Physics: Conference Series* **2009**, *190*, 012143.

(24) Stepanow, S.; Miedema, P. S.; Mugarza, A.; Ceballos, G.; Moras, P.; Cezar, J. C.; Carbone, C.; de Groot, F. M. F.; Gambardella, P. Mixed-valence behavior and strong correlation effects of metal phthalocyanines adsorbed on metals. *Physical Review B* **2011**, *83*, 220401.

(25) Lever, A. B. P. 336. The magnetic behaviour of transition-metal phathalocyanines. *Journal of the Chemical Society (Resumed)* **1965**, 1821–1829.

(26) Lee, S.; Yudkowsky, M.; Halperin, W. P.; Ogawa, M. Y.; Hoffman, B. M. One-dimensional magnetism in copper phthalocyanine. *Phys. Rev. B* **1987**, *35*, 5003–5007.

(27) Gargiani, P.; Rossi, G.; Biagi, R.; Corradini, V.; Pedio, M.; Fortuna, S.; Calzolari, A.; Fabris, S.; Cezar, J. C.; Brookes, N. B.; Betti, M. G. Spin and orbital configuration of metal phthalocyanine chains assembled on the Au(110) surface. *Phys. Rev. B* **2013**, *87*, 165407.
